# A revised digestion method to characterize manganese content in solids

**DOI:** 10.1016/j.mex.2024.102731

**Published:** 2024-04-22

**Authors:** Jérôme Ducret, Benoit Barbeau

**Affiliations:** Department of Civil, Geological and Mining Engineering, Polytechnique Montreal, 2500 chemin de Polytechnique, H3T 1J4, Montreal, QC, Canada

**Keywords:** Digestion methods, Manganese oxides, Metals content, Filter media, Distribution system, Concentrated HCl digestion method

## Abstract

Quantifying manganese (Mn) content in solids is critical for understanding its roles in aquatic ecosystems, soils, water treatment plants and distribution systems. No studies have yet used standard Mn oxides to compare the performance of the numerous digestion methods found in the literature. Nine digestion methods (including USEPA 3050B) were compared using four Mn oxides with varying oxidation states. The HCl concentrate (12.4 M) heated to at least at 40 °C provided quantitative digestion of all Mn oxides tested with ≈ 100 % recovery. HCl concentration is important only for MnO_2_ digestion, while temperature influences both MnO and MnO_2_ recovery. Complete recovery of various Al, Cu and Fe standard oxides using a 12.4 M HCl digestion at 95 °C. Digestion of environmental samples for Al, Ca, Fe, Mg and Mn content yielded higher metal content using the HCl method (except for Al). HCl 12.4 M digestion provided better performance than other digestion methods found in the scientific literature because of its high reducing capacity.

• Most digestion methods found in the literature do not digest all Mn oxidation states.
• Hydrochloric acid is shown to be essential to dissolve all oxidation state of Mn oxides.

## Graphical abstract

**Figure:**
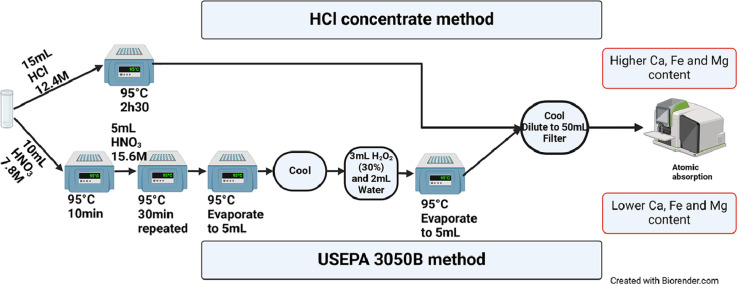

Specifications table

**Table:** 

Subject area:	*Environmental science*
More specific subject area:	Metal quantification
Name of your method:	Concentrated HCl digestion method
Name and reference of original method:	*US EPA Method 3050B – Acid digestion of sediments sludges and soil*
Resource availability:	Digestion system (HotBlock® SC100 for example) HCl concentrate (ACS grade)

## Background

Research on manganese (Mn) fate in water (Mn biogeochemistry in marine environment [1], impact of Mn in the distribution networks [2,3], Mn removal in water treatment [4], [5], [6] etc.) commonly requires a quantitative assessment of Mn content in solid deposits. To study manganese oxide accumulation in water systems, it is important to adopt a method that can quantitatively digest Mn with different natural oxidation states (e.g., Mn(+II), Mn(+III) and Mn(+IV)) given that each of them has been identified on biofilter media coatings [4,6]. This objective is normally achieved by performing an acid digestion of the solids followed by Mn analysis using either inductively coupled plasma (ICP) or atomic absorption (AA). Although elemental analysis of Mn using AA or ICP is standardized, numerous digestion methods have been proposed to dissolve manganese oxides (MnOx) (the most common ones are listed in Table S1). No comparison has yet been made on the performance of these various digestion methods, and there is no evidence that they can fully dissolve all types of naturally occurring MnOx deposits, as reported by Cerrato, et al. [7]. MnOx deposits are known to be stable in the environment (the thermodynamic equilibrium constants for some common Mn oxides at different average oxidation states (AOSs) are presented in Table S2). For a given AOS, Mn oxides are at least 17 orders of magnitude more stable (by comparing K_sp_) than their hydroxides counterpart. Therefore, it is important that any digestion method proposed to characterize MnOx considers the challenge imposed by manganese oxides.

Similarly, it has been shown that the United States Environmental Protection Agency (USEPA) standard digestion protocol was not fully effective in recovering particulate Pb [8,9]. A hypothesis was made that, similar to the challenge of lead oxide digestion, an optimized protocol is required to correctly characterize MnOx content in solid media, in particular for Mn(IV) oxides for which a reducer is needed as reagent. The USEPA 3050B standard solid digestion method uses HNO_3_ and H_2_O_2_ [10]. However, HNO_3_ is an oxidant in which MnO_2_ is not soluble [11], while MnO_2_ is known to catalyze H_2_O_2_ decomposition in water [12].

The aim of this study is to compare various digestion protocols to identify the most suitable technique to quantify Mn content in solids with mixed Mn oxidation states. Furthermore, the optimal method was compared to USEPA method 3050B for the characterization of reference solids as well as deposits collected from drinking water treatment plants and water distribution networks given that the optimal method should also be proficient for characterizing other common elements such as Al, Fe, Ca and Mg.

## Method details

**Fig. 1:**
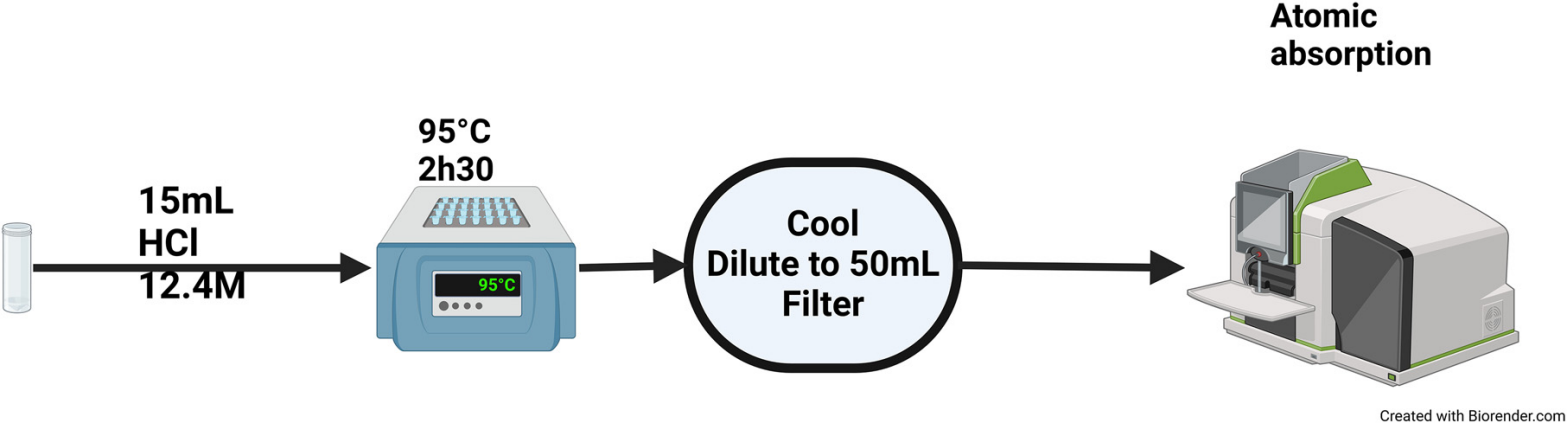
Presentation of the optimized digestion method for manganese oxides.

The digestion method to determine metal (in particular Mn) content in solids (tested on reference oxides and on environmental water treatment, distribution solids), is presented in the Fig. 1. The concentrated HCl method, which was determined to be the optimum method is described below.

Approximately 0.5 g of dry solids were weighed out and put into a digestion tube (acid and heat resistant). Then 15 mL of concentrate HCl (12.4 M) were added in the tube at room temperature. Digestion of the solids was done for 2.5 h at 95 °C under a chemical hood in a HotBlock® digestion system with a ribbed watch glass to limit evaporation. Samples were then cooled to room temperature for approximately 1 h and then diluted with Milli-Q water to a volume of 50 mL followed by a 2 µm filtration to remove insoluble materials (in particular silica particles). Metal quantification in the liquid phase was performed by atomic absorption. The detection limit of this method depends on the quantity of solid introduced and the quantification method used; within the framework of this article was to 0.001 mg Mn/g of dry media.

**Fig. 2:**
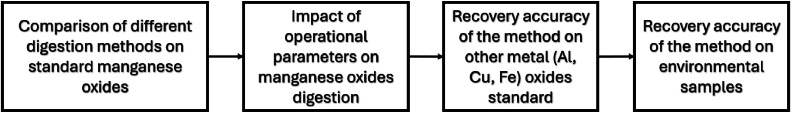
The different steps used in this study to validate the optimal digestion method for manganese oxides.

The recovery accuracy of this method was determined with the digestion of four manganese oxides standards with different average oxidation state and with other metal (Al, Cu and Fe) oxides standards and various environmental samples. The procedure followed for the characterization of the method is presented in Fig. 2.

## Method validation

### Reagents and chemicals

Reagents were purchased from Fisher Scientific (HNO_3_, Trace Metal Grade, oxalic acid dehydrate, ACS Grade, H_2_SO_4_, ACS Plus Grade), VWR (HCl 36.5–38 %, ACS Grade) and Sigma‒Aldrich (H_2_O_2_, ACS Grade). The digestion methods were tested on four reference manganese oxides (Sigma‒Aldrich, USA) confirmed by XRD (spectra are presented on Figure S1) as pyrolusite (MnO_2_, 99 % purity), bixbyite (Mn_2_O_3_, 99 % purity), hausmannite (Mn_3_O_4_, 97 % purity) and manganosite (MnO, 99 % purity). They were chosen as MnOx representative of different oxidation states expected to be found in water treatment solids.

All dilution and dissolution assays were conducted using ultrapure water from a Milli-Q integral water purification system (Millipore, USA). All plastic ware used was metal-free, and all glassware was soaked overnight in a 10 % (by volume) nitric acid solution and then rinsed three times with deionized water and three times with Milli-Q water.

### Statistical analysis

Kruskal‒Wallis tests, nonparametric analyses of variance [13], were conducted in R to compare EPA3050B with the 12.4 M HCl method and optimize the digestion conditions of the HCl method.

### Comparison of different digestion methods on standard manganese oxides

In total, nine digestion methods (presented in Table 1) were tested for their ability to dissolve the four standard manganese oxides presented above. Duplicate samples of 0.5 g solids were digested in 50-mL metal-free polypropylene cups (UC475, Delta Scientific), which were covered with a disposable watch glass (SC505, Delta Scientific) and heated in a 36-well HotBlock™ (SC100, Environmental Express, Delta Scientific). Negative controls were included in the experimental plan. At the end of the digestion period, the samples were diluted with ultrapure water and then filtered through a 2 µm composite PTFE-polypropylene membrane (Filtermate™, SC0401, Delta Scientific). Filtered samples were immediately analyzed for Mn (with a limit of quantification of 10 µg Mn/L) using flame atomic absorption (PinAAcle™ 900F, PerkinElmer, USA).

**Table 1:** Presentation of the different digestion methods tested.

Methods	Description	Associated references
EPA3050B	Step 1: 10 mL HNO_3_ (7.8 M), *T* = 95 °C, 10 min Step 2: 5 mL HNO_3_ (15.6 M) + evaporate to 5 mL, *T* = 95 °C, 2 h30 min Step 3: 2 mL Ultrapure Water + 3 mL H_2_O_2_ (30 %), Room temperature, 1 h Step 4: Evaporate to 5 mL, *T* = 95 °C	United States Environmental Protection Agency (USEPA) [10]
Aqua Regia	5 mL HNO_3_ (7.7 M) + 15 mL HCl (12.4 M), *t* = 95 °C, 2 h 30 min	No references
HCl concentrate (12.4 M)	15 mL HCl (12.4 M), *T* = 95 °C, 2 h 30 min	Almquist, et al. [14]; Tali [15]
HCl (4.1 M) + 2 g/L C_2_H_2_O_4_	30 mL HCl (4.1 M) + 2 g/L C_2_H_2_O_4_, *T* = 95 °C, 2 h 30 min	Breda, et al. [16]; de Vet, et al. [17]
HCl (6.2 M) + 2 g/L C_2_H_2_O_4_	30 mL HCl (6.2 M) + 2 g/L C_2_H_2_O_4_, *T* = 95 °C, 2 h 30 min	No references
HCl (12.4 M) + 2 g/L C_2_H_2_O_4_	30 mL HCl (12.4 M) + 2 g/L C_2_H_2_O_4_, *T* = 95 °C, 2 h 30 min	No references
H_2_SO_4_ (0.5 M) + 45 g/L C_2_H_2_O_4_	30 mL H_2_SO_4_ (0.5 M) + 45 g/L C_2_H_2_O_4_, *T* = 95 °C, 2 h 30 min	Kijima, et al. [18]
H_2_SO_4_ (17.6 M)	15 mL H_2_SO_4_ (17.6 M), *T* = 95 °C, 2 h 30 min	No references
HNO_3_ (15.4 M)	Step 1: 10 mL HNO_3_ (7.7 M), *T* = 95 °C, 30 min Step 2: 5 mL HNO_3_ (15,4 M), *T* = 95 °C, 2 h 30 min	No references

The main differences between the various digestion methods relate to (i) the types of acid used (H_2_SO_4_, HCl, HNO_3_, oxalic acid or a mix), (ii) their concentrations, (iii) the duration of the experiment (2 h 30 vs. more than 4 h) and (iv) the number of steps (1 to 4). The most tedious method is the standard method USEPA 3050B which involves four steps. All methods involved incubating the sample at 95 °C. Oxalic acid is a dicarboxylic acid that is known for its ability to sequester metals caused by its high reduction capacity.

The performance of nine digestion methods to recover manganese from four types of MnOx (Table 2) was assessed. Generally, pyrolusite proved to be the most difficult MnOx to digest, and only two methods (HCl concentrate (12.4 M) and EPA3050B) provided an overall recovery level of above 90 % for all types of MnOx.

**Table 2:** Comparison of manganese recovery (average +/- std deviation) achieved by nine digestion methods for four standard manganese oxides.

Oxide	Manganosite	Hausmannite	Bixbyite	Pyrolusite	Overall recovery
Formula	MnO	Mn_3_O_4_	Mn_2_O_3_	MnO_2_	
Oxidation State	+II	+II, +III	+III	+IV	–
Particle size (µm)	250	5.3	6.6	71	–
EPA3050B	99 ± 5 %	100 ± 3 %	102 ± 3 %	99 ± 31 %	100 ± 8 %
Aqua regia	96 ± 1 %	92 ± 1 %	65 ± 12 %	76 ± 10 %	82 ± 4 %
HCl (12.4 M)	105 ± 3 %	103 ± 5 %	105 ± 2 %	98 ± 0.4 %	102 ± 2 %
HCl (4.1 M) + 2 g/L C_2_H_2_O_4_	84 ± 15 %	97 ± 0.2 %	93 ± 1 %	12 ± 1 %	72 ± 4 %
HCl (6.2 M) + 2 g/L C_2_H_2_O_4_	93 ± 5 %	95 ± 0.4 %	92 ± 7 %	68 ± 1 %	87 ± 2 %
HCl (12.4 M) + 2 g/L C_2_H_2_O_4_	91 ± 5 %	81 ± 15 %	92 ± 8 %	82 ± 2 %	87 ± 4 %
H_2_SO_4_ (0.5 M) + 45 g/L C_2_H_2_O_4_	71 ± 16 %	59 ± 7 %	64 ± 6 %	91 ± 9 %	72 ± 5 %
H_2_SO_4_ (17.6 M)	2 ± 2 %	3 ± 2 %	6 ± 0.5 %	1 ± 1 %	3 ± 1 %
HNO_3_ (15.4 M)	69 ± 8 %	35 ± 10 %	31 ± 2 %	0 ± 0.02 %	34 ± 3 %

The EPA 3050B method is recommended for the digestion of sediments [10]. However, this technique requires multiple steps and yields more variability in MnO_2_ recovery (±31 %). Although the USEPA and concentrated HCl (12.4 M) methods did not provide significantly different results (*p* > 0.05), we selected the use of HCl (12.4 M) as a superior method given its simplicity and its lower variability regarding MnO_2_ recovery. In addition, all other MnOx can be effectively dissolved by HCl (Table 2) [11].

## Method optimization and performance evaluation

Once the use of HCl was identified as the best acid for Mn digestion, the impact of temperature and HCl concentration on Mn recovery was assessed for each standard Mn oxide studied. First, triplicate digestions were performed with HCl 12.4 M at 30, 40, 70 and 95 °C. Secondly, standard Mn oxides were digested in triplicate at 95 °C with HCl concentrations of 4.1, 6.2 or 12.4 M.

Once the optimal method (HCl 12.4 M at 95 °C) was identified, its performance was measured by calculating Mn recovery from samples analyzed in triplicate. Performance was compared to that of the standard method EPA3050B, which was also tested in triplicate.

## Optimization of concentrate HCl method for the recovery of Mn from MnOx

**Fig. 3:**
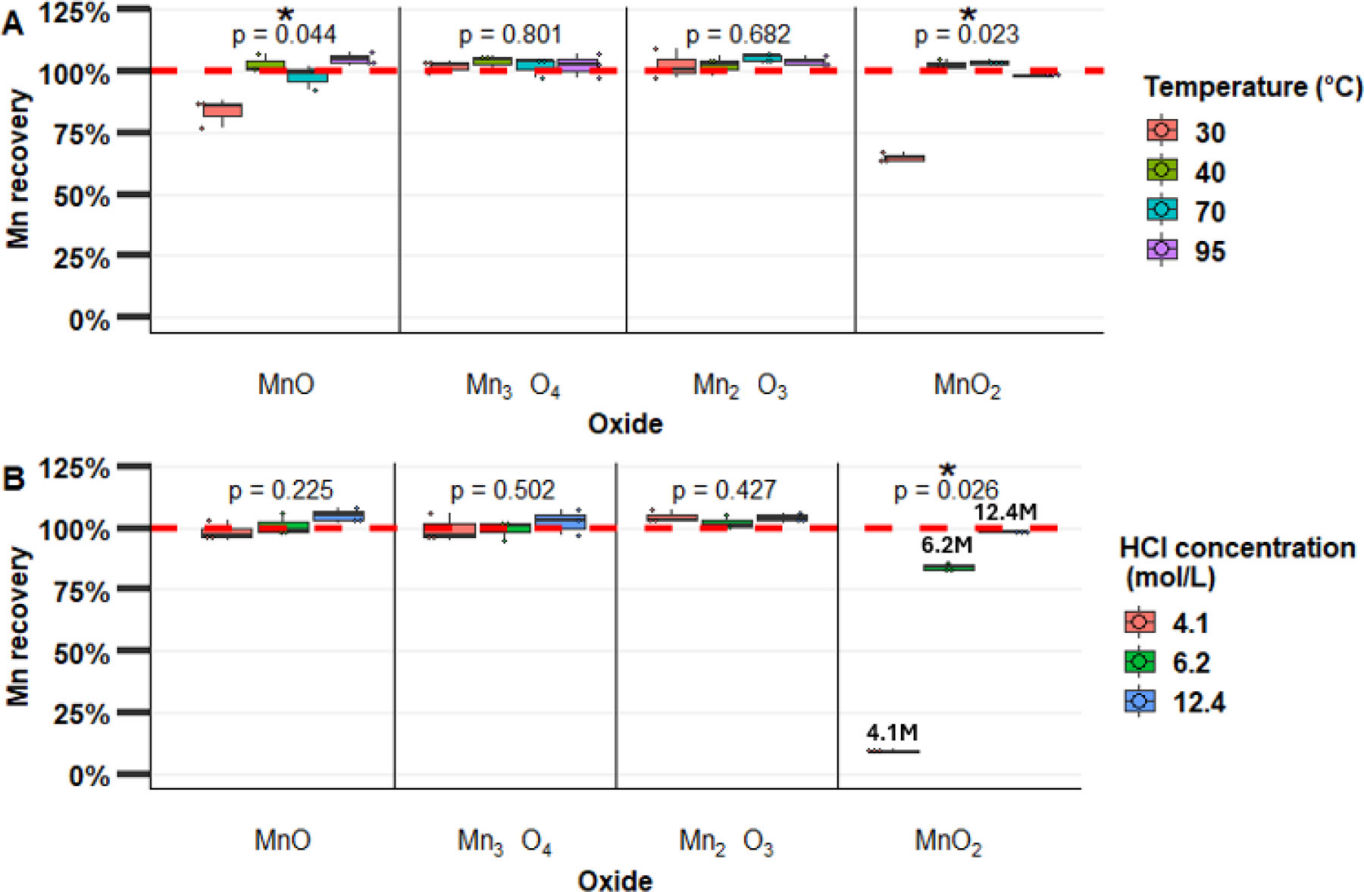
Mn recovery for four Mn standard oxides using the HCl digestion method depending on (A) digestion temperature (using 12.4 M HCl) and (B) HCl concentration (at 95 °C).

The impact of temperature on digestion performance was tested by comparing MnOx recoveries at 30, 40, 70 and 95 °C using 12.4 M HCl (Fig. 3A). The boiling temperature of HCl 12.4 M is approximately 50 °C [19]. These results show that for temperatures higher than 40 °C, all Mn oxides were effectively digested, which implies that it is not necessary to exceed boiling when using 12.4 M HCl. Further digestions were performed at 95 °C to help dissolve other metal oxides.

Mn recovery was observed to be dependent on HCl concentration (*p* < 0.05, Fig. 3B). To maximize the recovery of MnO_2_, it is recommended to use HCl without dilution (i.e., HCl 12.4 M).

## Impact of particle size on digestion performance

A sample of pyrolusite (Manganox™, Magnus, Canada) was sifted to obtain three size fractions of different diameters (d) with a uniformity coefficient of < 1.65: (i) 0.35 mm ≤ *d* ≤ 0.50 mm, (ii) 0.50 mm ≤ *d* ≤ 0.71 mm, and (iii) 0.71 mm ≤ *d* ≤ 0.85 mm. The different fractions were digested in triplicate with the HCl concentrate method.

The digestion of different pyrolusite size fractions showed that particle sizes did not significantly (*p* > 0.05) impact digestion performance by the HCl concentrate (12.4 M) method (data not shown).

## Recovery accuracy of the method on other metal (Al, Cu and Fe) oxides standard

The optimized digestion method was tested to digest other metal oxides (purchased from Sigma‒Aldrich, USA) that have been commonly found in drinking water systems, such as Fe_3_O_4_ (95 % purity) [20], CuO (98 % purity) [21], Cu_2_O (97 % purity) [21] and Al(OH)_3_ (reagent grade) [22]. Fe, Al and Cu oxide standards were digested, filtered and immediately analyzed (with a limit of quantification of 20 µg Fe/L, 50 µg Cu/L and 1 mg Al/L) using flame atomic absorption (PinAAcle™ 900F, PerkinElmer, USA).

Table 3 presents the recovery of the digestion of the four standard oxides tested. For each standard oxide tested, the metal recovery level was 100 % for aluminum, copper, and iron. HCl concentrate (12.4 M) method can be used to digest solids to quantitatively determine metals other than Mn.

**Table 3:** Metal recovery for the digestion of four standard oxides (traditionally present in drinking water systems) with the HCl method.

Oxide	Metal average oxidation state in solids	Metal recovery
Al(OH)_3_	3	100 ± 1 %
CuO	2	103 ± 3 %
Cu_2_O	1	100 ± 1 %
Fe_3_O_4_	2.67	100 ± 2 %

### Recovery accuracy of the method on environmental samples

Manganese content was tested on four virgin materials commonly used in water filtration (anthracite, activated carbon, sand and Filtralite™) as a negative control of the HCl method. Negative controls were found to present Mn content below the detection limit (< 0.001 mg Mn/g of dry media).

To validate the applicability of the HCl method for characterizing natural samples, filter media were recovered from full-scale water treatment plants before and after backwashing. Metal content (Mn, Fe, Ca, Mg and Al) was quantified using the optimized HCl and EPA3050B methods. All assays were conducted in triplicate. The first filter media sample was collected on the anthracite surface of a dual media (anthracite greensand) supplemented with continuous chlorine and potassium permanganate regeneration for Mn removal from groundwater. The second medium originates from a groundwater system equipped with catalytic pyrolusite filters with intermittent chlorine regeneration. The third sample was collected from the top layer (anthracite) of a dual media granular filter (sand/anthracite) supplemented with coagulated-settled-chlorinated surface water operating as a catalytic filter to remove 0.1–0.2 mg Mn/L present in the feed water during the summer. The last media originates from a maturing sand biofilter supplemented with Mn- and Fe-contaminated groundwater for six months. For each metal, the relative bias between the HCl and EPA3050B methods was calculated using Eq. (1).

(1) $$Bias\;\left(\%\right)=\;\frac{HCl\;metal\;content-EPA3050B\;metal\;content}{HCl\;metal\;content}\#\;\times \;100$$

Unidirectional flushing is a common maintenance activity in distribution systems. Fire hydrants are opened in a manner that promotes high velocity, which scours the area inside the pipes. Ten samples were collected during unidirectional flushing on a Canadian utility supplemented with groundwater exhibiting iron and manganese contamination and a total hardness of 200 mg CaCO_3_/L.

The solid content of each sample was harvested by filtration using a 2 µm filter (RAWG04700, MF-Millipore™) and then dried at 60 °C (the filter does not support heating at temperatures greater than 75 °C) for 48 h. As these filters are made from nitrocellulose membrane, they are fully dissolved in concentrated nitric acid or hydrochloric acid. Each sample was then digested in duplicate with the optimized HCl and EPA3050B methods. For each metal, the bias between both methods was calculated using Eq. (1).

**Fig. 4:**
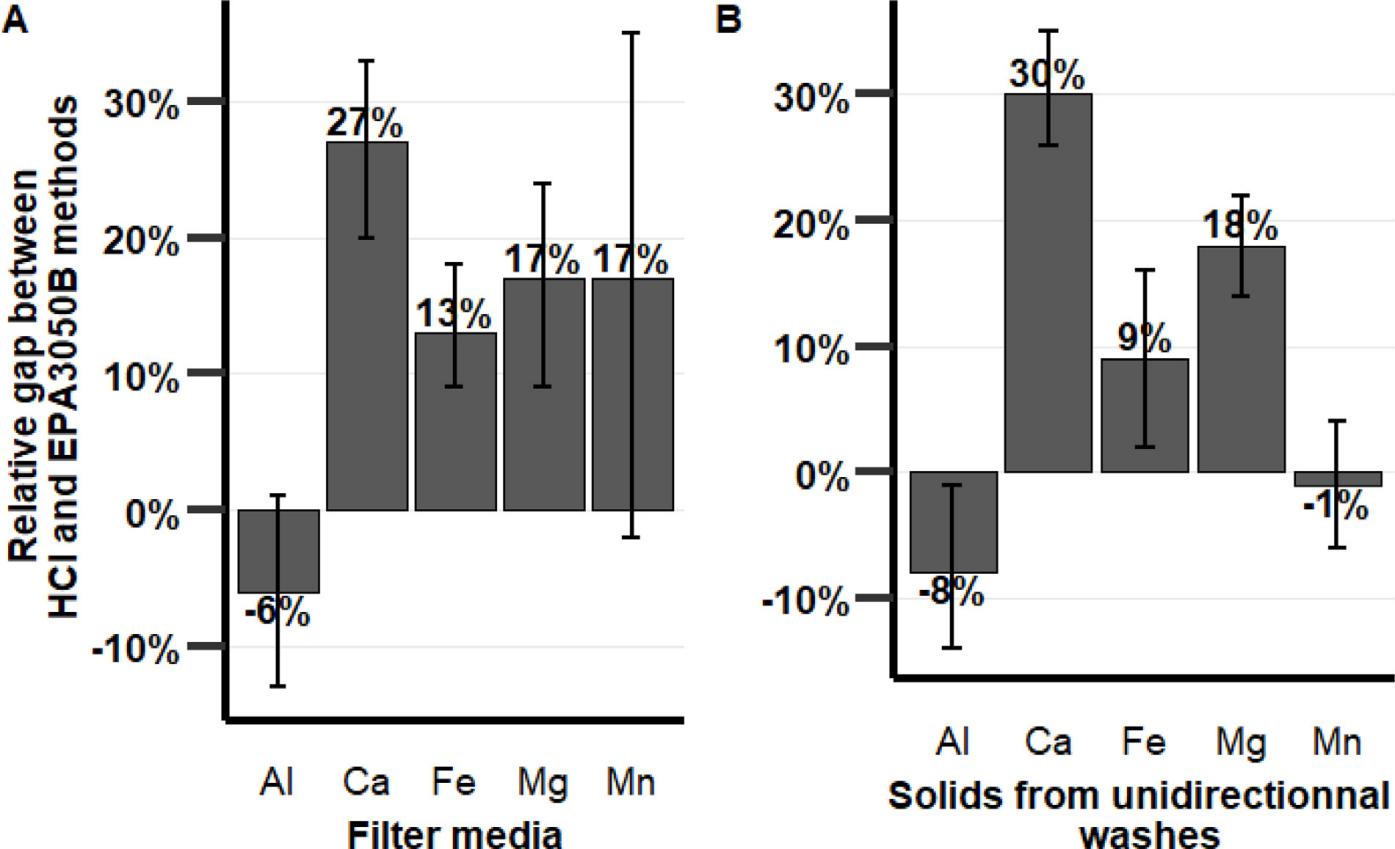
Metals (Al, Ca, Fe, Mg, and Mn) content comparison using the two digestion methods of media coatings from four different filters. The error bars represent the standard deviation calculated for each triplicate sample.

Fig. 4 presents the mineral (Al, Ca, Fe, Mg, and Mn) content bias observed between the HCl and EPA3050B methods for the digestion of filter media (A) and for solids harvested from unidirectional flushing waters (B). Both methods provided statistically equivalent (*p* > 0.05) aluminum content. For the Mn content, filter media digested by the HCl method recovered 17 ± 19 % more Mn than EPA3050B (*p* > 0.05), while for solids from unidirectional flushing, the Mn contents were equivalent. The difference observed might be explained by the Mn oxidation state in the solids (+4 or closer of 4 for filter media and less for solids from unidirectional flushing). Calcium, iron, and magnesium levels were statistically higher (*p* < 0.05) when using the HCl method than when using the EPA3050B method. The HCl method therefore provided more recovery than the EPA3050B method for the environmental samples investigated. EPA3050B has been tested on natural samples for some minerals (Ag, As, Ba, etc.) [10], but none of the minerals tested in the current study were used to validate the efficacy of the USEPA3050B method. This probably explains why the low recovery of Ca, for example, has not been highlighted in the scientific literature.

Given its performance and simplicity, we recommend using concentrated HCl digestion to characterize environmental MnOx samples, especially if the samples are expected to include solids with high average Mn oxidation states (e.g., pyrolusite).

## Limitations

For ICP-MS analysis, it is important to be careful about chloride related polyatomic interferences (e.g. arsenic and vanadium) [23] or interfenrences caused by high levels of total dissolved solides caused by the used of concentrate HCl [24].

## Ethics statements

None.

## CRediT authorship contribution statement

**Jérôme Ducret:** Conceptualization, Methodology, Software, Formal analysis, Investigation, Writing – original draft, Visualization. **Benoit Barbeau:** Conceptualization, Methodology, Formal analysis, Resources, Writing – review & editing, Supervision, Project administration, Funding acquisition.

## Declaration of competing interest

The authors declare that they have no known competing financial interests or personal relationships that could have appeared to influence the work reported in this paper.

## Data Availability

Data will be made available on request.

## Appendix Supplementary materials

Supplementary material and/or additional information Supplementary materials about the thermodynamic equilibrium constant of different manganese and a list of the different digestion methods used in the literature are available online. Image, application 1
