# Supplementary material for: A revised digestion method to characterize manganese content in solids
Source: MethodsX. 2024 Apr 22;12:102731. doi: 10.1016/j.mex.2024.102731 (PMC11068846; doi:10.1016/j.mex.2024.102731)
Supplement: Supplementary file 1 — Supplementary material and/or additional information Supplementary materials about the thermodynamic equilibrium constant of different manganese and a list of the different digestion methods used in the literature are available online. [file mmc1.docx]

**Supplementary materials:**

1. **Examples of digestion methods used in the literature to characterize Mn content in solids**

Table S1: Examples of digestion methods formerly used to characterize Mn content in solids.

| **Chemicals used** | **Temperature (°C)** | **Incubation time**  **(h)** | **References** |
| --- | --- | --- | --- |
| 0.2 M oxalic acid + 0.2 M ammonium oxalate | NS | overnight | Albers, et al. [1] |
| 4 M HCl + 2 g/L oxalic acid | NS | NS | Breda, et al. [2]; de Vet, et al. [3] |
| 3 M HNO_3_ | Boiling | NS | Bruins, et al. [4] |
| 9.3 M HNO_3_ | 40 °C | 30 min + 30 min sonication | Burger, et al. [5] |
| 7.7 M HNO_3_ + 15.4 M HNO_3_ + H­_2_O_2_ (30%) (optional: +HCl) | 95 °C | 4 h | McCormick, et al. [6]; United States Environmental Protection Agency (USEPA) [7] |
| 12.4 M HCl | 80 °C | NS | Almquist, et al. [8]; Tali [9] |
| 0.5 M H_2_SO_4_ + 0.5 M oxalic acid | NS | NS | Kijima, et al. [10] |
| 0.074 M HNO_3_ + 6 g/L hydroxylamine sulfate | NS | 2 h | Cerrato, et al. [11]; Knocke, et al. [12] |
| 0.148 M HNO_3_ + 4 g/L hydroxylamine sulfate | NS | 6 h | Tobiason, et al. [13]  Islam, et al. [14] |
| 0.01M HNO_3_ + 0.1M NH_2_OH.HCl | Room Temperature | 0.5h | Chao [15] |

*NS: not specified*

1. **Manganese oxide thermodynamic solubility constant calculation:**

Table S2: Thermodynamic data of common manganese oxides and hydroxides

| **Minerals** | **Formula** | **AOS** | **ΔG°_f_ (kj/mol)** | **K_sp_** | **Source** |
| --- | --- | --- | --- | --- | --- |
| Pyrochroite | Mn(OH)_2_ | +II | -615.71 | 1.5×10^-13^ | Hem et Lind [16] |
| Hausmannitte | Mn_3_O_4_ | (+II,+III) | -1283.34 | 1.0×10^-119^ |  |
| Bixbyte | Mn_2_O_3_ | +III | -881.16 | 1.6×10^-34^ | Hem [17] |
| Groutite | α-MnOOH | +III | -556.08 | 1.6×10^-13^ | Fritsch, et al. [18]; Sun, et al. [19] |
| Feitknechtite | β-MnOOH | +III | -547.11 | 4.0×10^-17^ | Sun, et al. [19] |
| Manganite | γ-MnOOH | +III | -557.72 | 1.6×10^-15^ | Hem et Lind [16] |
| Nsutite | γ-MnO_2_ | +IV | -461.91 | Insoluble* | Kitchaev, et al. [20] |
| Ramsdellite | R-MnO_2_ | +IV | -460.00 | Insoluble* |  |
| K-Birnessite | δ-(K,Mn)O_2_ | +IV | -580.64 | Insoluble* | Birkner et Navrotsky [21] |
| Na-Birnessite | δ-(Na,Mn)O_2_ | +IV | -556.00 | Insoluble* |  |
| Ca-Birnessite | δ-(Ca,Mn)O_2_ | +IV | -546.65 | Insoluble* |  |
| Pyrolusite | β-MnO_2_ | +IV | -465.19 | Insoluble* | Hem et Lind [16] |

*The calculation method is presented below. The thermodynamic equilibrium constant (K_sp_) was calculated at 25°C and atmospheric pressure. *Mn(IV) oxides are completely insoluble, so no K_sp_ can be calculated for these species without considering a reduction of Mn(IV) to Mn(III) or Mn(II) [22, 23].*

For each Mn oxide considered, the chemical equation used for the calculations is presented in Table S3. The equations are balanced for a basic environment to trace the main pH found in biological water treatment for manganese removal.

Gibbs energy from the solubility equation has been calculated using Equations S1.1 and S1.2 using for each component their Gibbs energy of formation taken from NBS table [23], except for manganese oxides or hydroxides, for which this value depends on their mineralogy arrangement [19, 21]. Then, the solubility constant is determined using Equation S1.3.

$$\begin{aligned} \alpha A+\beta B\underset{\leftrightarrow}{h}\gamma C+\delta D\#\left( S1.1 \right) \end{aligned}$$

With (α,β,γ,δ): stoichiometric coefficients

$$\begin{aligned} {\Delta G^{\circ}}_{f}={\gamma\Delta_{f}G^{\circ}}_{C}+{\delta\Delta_{f}G^{\circ}}_{D}-\left( {\alpha\Delta_{f}G^{\circ}}_{A}+{\beta\Delta_{f}G^{\circ}}_{B} \right)\#\left( S1.2 \right) \end{aligned}$$

$$\begin{aligned} K_{sp}=e^{\frac{-{\Delta G^{\circ}}_{f}}{RT}}\#\left( S1.3 \right) \end{aligned}$$

*Table S3: Chemical reaction for the solubility of Mn oxides presented in this study.*

| **Manganese oxides** | **Solubility equilibria considered** |
| --- | --- |
| Pyrochroite [Mn(OH)­_2_] | ${Mn(OH)}_{2}\underset{\leftrightarrow}{f}{Mn}^{2+}+2{HO}^{-}$ |
| Hausmannite [Mn_3_O_4_] | ${Mn}_{3}O_{4}+{4H}_{2}O\underset{\leftrightarrow}{f}{Mn}^{2+}+2{Mn}^{3+}+4{HO}^{-}$ |
| Bixbyite [Mn_2_O_3_] | ${Mn}_{2}O_{3}+{3H}_{2}O\underset{\leftrightarrow}{f}2{Mn}^{3+}+6{HO}^{-}$ |
| Groutite [α-MnOOH] | $\alpha-MnOOH+ H_{2}O\underset{\leftrightarrow}{f}{Mn}^{3+}+3{HO}^{-}$ |
| Feitknechtite [β-MnOOH] | $\beta-MnOOH+ H_{2}O\underset{\leftrightarrow}{f}{Mn}^{3+}+3{HO}^{-}$ |
| Manganite [γ-MnOOH] | $\gamma-MnOOH+ H_{2}O\underset{\leftrightarrow}{f}{Mn}^{3+}+3{HO}^{-}$ |

1. **XRD spectrums of the four reference manganese oxides**


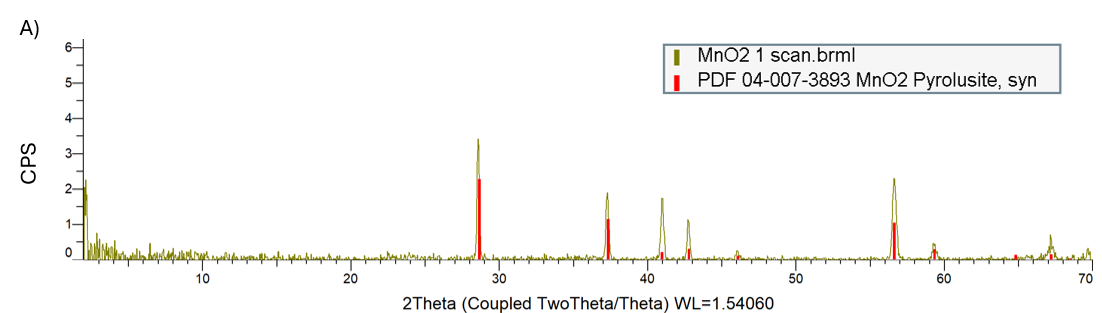


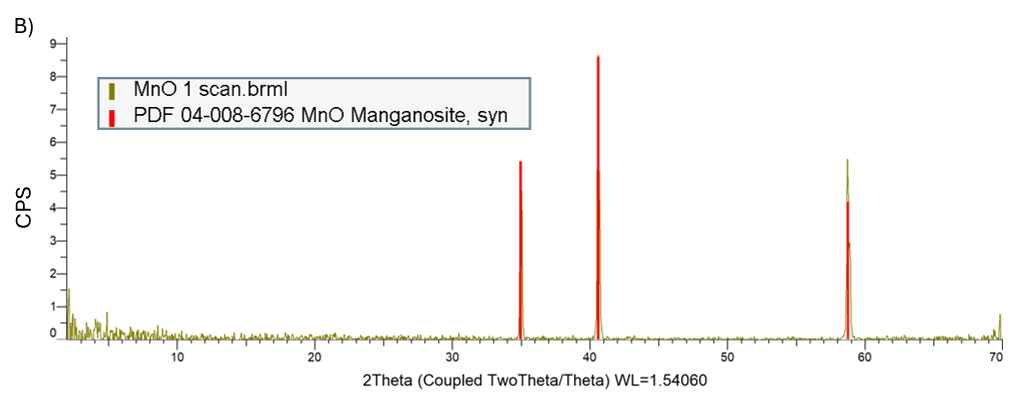


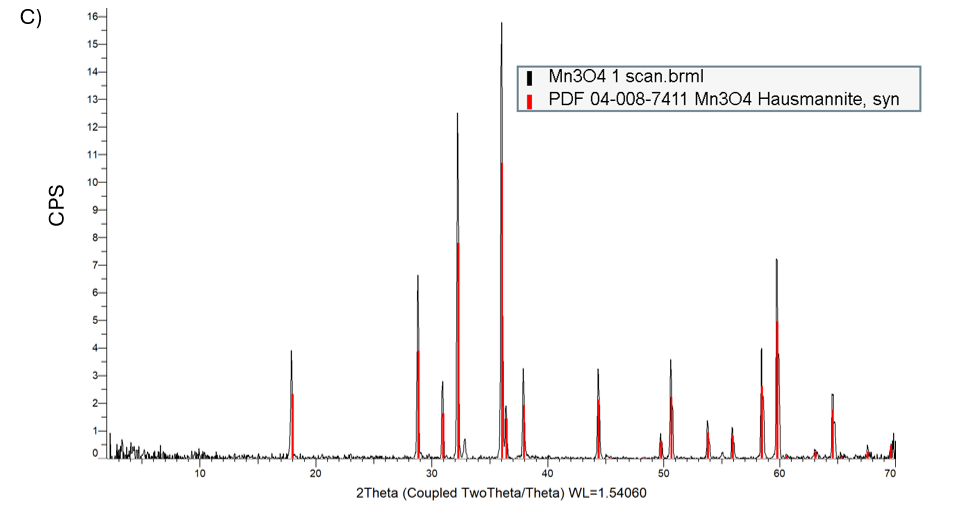


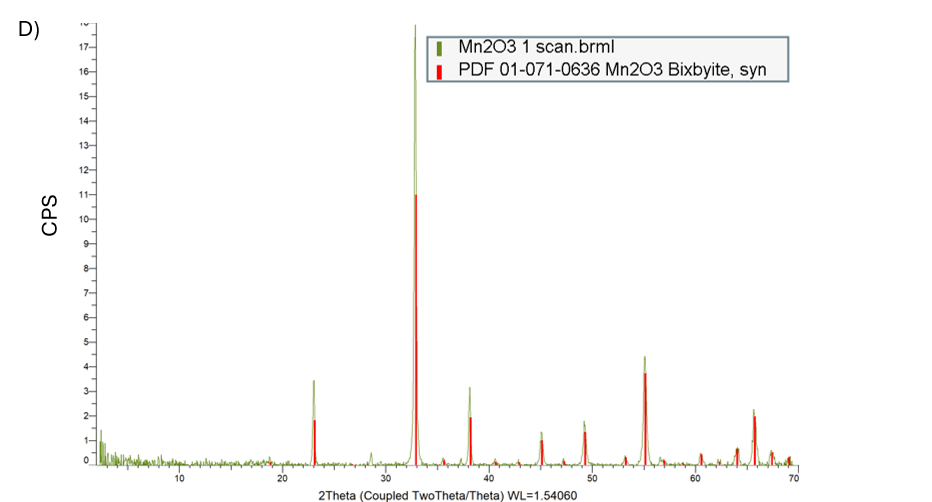


*Figure S1: XRD spectra used to determine the structure of (A) MnO_2_; (B) MnO; (C) Mn_3_O_4_ and (D) Mn_2_O_3_.*

**References**

1. Albers, C., et al., *Groundwater chemistry determines the prokaryotic community structure of waterworks sand filters.* Environmental science & technology, 2014. **49**.

2. Breda, I.L., et al., *Manganese removal processes at 10 groundwater fed full-scale drinking water treatment plants.* Water Quality Research Journal, 2019. **54**(4): p. 326-337.

3. de Vet, W.W.J.M., et al., *Assessment of nitrification in groundwater filters for drinking water production by qPCR and activity measurement.* Water Research, 2011. **45**(13): p. 4008-4018.

4. Bruins, J.H., et al., *Reduction of ripening time of full-scale manganese removal filters with manganese oxide-coated media.* Journal of Water Supply: Research and Technology-Aqua, 2015. **64**(4): p. 434-441.

5. Burger, M.S., et al., *Manganese removal during bench-scale biofiltration.* Water Research, 2008. **42**(19): p. 4733-4742.

6. McCormick, N.E., et al., *Understanding the impact of different source water types on the biofilm characteristics and microbial communities of manganese removing biofilters.* Environmental Science: Water Research & Technology, 2023. **9**(1): p. 48-61.

7. United States Environmental Protection Agency (USEPA), *Method 3050B - Acid digestion of sediments, sludges, and soils (Revision)*. 1996. p. 12.

8. Almquist, C., M. Krekeler, and L. Jiang, *An investigation on the structure and catalytic activity of cryptomelane-type manganese oxide materials prepared by different synthesis routes.* Chemical Engineering Journal, 2014. **252**: p. 249-262.

9. Tali, R., *Determination of average oxidation state of Mn in ScMnO3 and CaMnO3 by using iodometric titration.* Damascus Univ. J. Basic Sci., 2007. **23**: p. 9-19.

10. Kijima, N., et al., *Preparation and Characterization of Open Tunnel Oxide α-MnO2 Precipitated by Ozone Oxidation.* Journal of Solid State Chemistry, 2001. **159**(1): p. 94-102.

11. Cerrato, J.M., et al., *Use of XPS to Identify the Oxidation State of Mn in Solid Surfaces of Filtration Media Oxide Samples from Drinking Water Treatment Plants.* Environmental Science & Technology, 2010. **44**(15): p. 5881-5886.

12. Knocke, W.R., S.C. Occiano, and R. Hungate, *Removal of soluble manganese by oxide-coated filter media: sorption rate and removal mechanism issues.* Journal American Water Works Association, 1991. **83**: p. 64-69.

13. Tobiason, J.E., et al., *Characterization and performance of filter media for manganese control*. 2008, American Water Works Association Research Foundation: Denver, Colorado, USA. p. 306.

14. Islam, A.A., et al., *Characterization of filter media MnOx(s) Surfaces and Mn removal capabilit.* American Water Works Association, 2010. **102**(9): p. 71.

15. Chao, T.T., *Selective Dissolution of Manganese Oxides from Soils and Sediments with Acidified Hydroxylamine Hydrochloride.* Soil Science Society of America Journal, 1972. **36**(5): p. 764-768.

16. Hem, J.D. and C.J. Lind, *Nonequilibrium models for predicting forms of precipitated manganese oxides.* Geochimica et Cosmochimica Acta, 1983. **47**(11): p. 2037-2046.

17. Hem, J.D., *Redox processes at surfaces of manganese oxide and their effects on aqueous metal ions.* Chemical Geology, 1978. **21**(3): p. 199-218.

18. Fritsch, S., J.E. Post, and A. Navrotsky, *Energetics of low-temperature polymorphs of manganese dioxide and oxyhydroxide.* Geochimica et Cosmochimica Acta, 1997. **61**(13): p. 2613-2616.

19. Sun, W., et al., *Non-equilibrium crystallization pathways of manganese oxides in aqueous solution.* Nature Communications, 2019. **10**(1): p. 573.

20. Kitchaev, D.A., et al., *Thermodynamics of phase Selection in MnO2 framework structures through alkali intercalation and hydration.* Journal of the American Chemical Society, 2017. **139**(7): p. 2672-2681.

21. Birkner, N. and A. Navrotsky, *Thermodynamics of manganese oxides: Sodium, potassium, and calcium birnessite and cryptomelane.* Proceedings of the National Academy of Sciences, 2017. **114**(7): p. E1046-E1053.

22. Tebo, B.M., et al., *Biogenic manganese oxides: Properties and mechanisms of formation.* Annual Review of Earth and Planetary Sciences, 2004. **32**(1): p. 287-328.

23. Reed, J.J., *The NBS Tables of Chemical Thermodynamic Properties: Selected Values for Inorganic and C1 and C2 Organic Substances in SI Units*. 2020, National Institute of Standards and Technology.
